# Role of microbial laccases in valorization of lignocellulosic biomass to bioethanol

**DOI:** 10.3389/fbioe.2024.1441075

**Published:** 2024-07-23

**Authors:** Ikram ul Haq, Aroona Saleem, Rida Chaudhary, Abdulrahman H. Alessa, Ali Nawaz, Chenyu Du

**Affiliations:** ^1^ Institute of Industrial Biotechnology (IIIB), GC University Lahore, Lahore, Pakistan; ^2^ Department of Biology, Faculty of Science, University of Tabuk, Tabuk, Saudi Arabia; ^3^ School of Applied Sciences, University of Huddersfield, Huddersfield, United Kingdom

**Keywords:** biofuels, bioethanol, enzymatic hydrolysis, microbial laccases, detoxification, delignification, laccase mediator system

## Abstract

The persistent expansion in world energy and synthetic compounds requires the improvement of renewable alternatives in contrast to non-sustainable energy wellsprings. Lignocellulose is an encouraging feedstock to be utilized in biorefineries for its conversion into value-added products, including biomaterials, biofuels and several bio-based synthetic compounds. Aside from all categories, biofuel, particularly bioethanol is the most substantial fuel derived from lignocellulosic biomass and can be obtained through microbial fermentation. Generally, extreme settings are required for lignocellulosic pretreatment which results in the formation of inhibitors during biomassdegradation. Occasionally, lignin polymers also act as inhibitors and are left untreated during the pretreatment, engendering inefficient hydrolysis. The valorization of lignocellulosic biomass by laccases can be viewed as a fundamental trend for improving bioethanol production. However, one of the main obstacles for developing commercially viable biofuel industries is the cost of enzymes, which can be resolved by utilizing laccases derived from microbial sources. Microbial laccases have been considered an exceptionally integral asset for delignification and detoxification of pretreated LCB, which amplify the resultant fermentation and saccharification processes. This review provides a summary of microbial laccases and their role in valorizing LCB to bioethanol, compelling enthralling applications in bio-refining industries all across the globe.

## 1 Introduction

Renewable fuels are referred as a favorable substitute for global warming besides diminishing dependence on fossil fuels (Fillat et al., 2017). During the end of the 20th century, renewed interests have been developed in the utility of bioethanol and biodiesel (Srivastava et al., 2020). Bioethanol production as a substitute for gasoline is a major concern these days due to its burning speed, elevated antiknock rating, and vaporizing temperature. The traditional (1st generation) bioethanol production is from starch and sugar feedstocks yet, corn, sugarcane juices, and treacle are also being utilized (Mishra and Ghosh, 2016). Conversely, lignocellulosic biomass is perceived as a significant crude material for biofuels, particularly for advanced (2nd generation) bioethanol production (Lin and Lu, 2021) owing to its minimal cost (Rezania et al., 2020) and is also employed at commercial scale production of bioethanol, since back in 2013 (Su et al., 2020). Bioethanol has been proven to release lower greenhouse gas (GHG) emissions as compared to petroleum-based fuels, especially when using sustainable feedstocks and production methods. Studies have shown a reduction of GHG emissions by 12%–19% when using a 10% ethanol blend in gasoline compared to regular gasoline (Graham et al., 2008; Han et al., 2015). Bioethanol derived from certain sources like switchgrass can achieve over 60% reduction in GHG emissions compared to gasoline. In contrast to other fuels, bioethanol is environmentally benign, can be effortlessly stored and is also an alluring choice for the transport sector, consequently delineating its favorability over petroleum and other fuels. Likewise, every year, the bioethanol production rate is escalating globally on account of its vast applications in industrial, pharmaceutical, and cosmetic practices (Choo et al., 2019; Lecksiwilai and Gheewala, 2020).

Lignocellulosic raw materials used for producing bioethanol are usually cheap and cultivable, which ultimately makes the process cost-effective and beneficial for the local economy (Bayrakci Ozdingis and Kocar, 2018). Biochemical changing of lignocellulose addresses the most ideal course among all scientific advancements (Fillat et al., 2017) and involves three significant steps; pretreatment enzymatic hydrolysis, and fermentation (Chen and Liu, 2015; Liu and Chen, 2016). Chemicals (e.g., hydrochloric acid, phosphoric acid, and hydrogen sulfide), biofuels (such as bioethanol, bio-hydrogen, and bio-butanol), and other biopolymers (like lignocellulosic biopolymers) produced as a result of pretreatment participates in the biochemical transformation towards disturbing the firmly related arrangements of lignin, cellulose, and hemicellulose molecules (Kim, 2018). While, during enzymatic hydrolysis, monosaccharides created by the hydrolysis of polysaccharides eventually transformed into ethanol through the process of fermentation by utilizing several microorganisms (Chen and Liu, 2017). Pretreatment is referred as an elementary step for LCB valorization. Yet, despite all its assets, it also exhibits some restrictions such as pretreatment of LCB is not an ecofriendly method as it entails traditional procedures of heating and employment of strong chemicals (Hassan et al., 2018). The process also results in the production of various inhibitors against several microbes along with other enzymes which hinders the fermentation process and ethanol generation, implicating a gridlock for the production of bioethanol (Jönsson and Martín, 2016).

**FIGURE 1:**
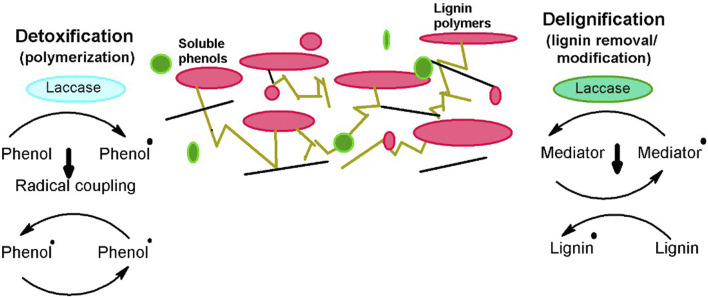
Role of lacasses for efficient conversion of biomass into bioethanol (Fillat et al., 2017).

Laccases are frequently utilized for valorizing the LCB and are much vital approach for bioethanol production as the methodology is convenient and also supports the viability at a commercial scale. Laccases are unique oxidative enzymes that produce two water molecules by the reduction of oxygen being their only co-substrate (Hashmi et al., 2016). They are widely utilized in enhancing bioethanol production by efficiently taking off the inhibitory contents of pretreated biomass via delignification and detoxification processes (Moreno et al., 2020), playing a central part in lignin modification and degradation. Moreover, the extended specificity of laccases for substrate makes them appropriate for industrial use as well (Agrawal et al., 2018). According to the international laccase market research report in 2018, overall laccase consumption has been increased to 166 MT from 140.75 MT in just the past 3 years. There are various means by which laccases can be obtained, i.e., plants, animals (Janusz et al., 2020), and microbes. However, microbial laccases are extensively preferred as it is a cheap source, make the process economical, and competently oxidize the toxic and non-toxic substrates. Microbial laccases are usually produced from fungal or bacterial species and are widely employed for the detoxification and delignification of different pretreated and processed un-pretreated feedstocks (Figure 1), either alone or with a laccase mediator system (LMS) (Bayrakci Ozdingis and Kocar, 2018). This review aims to provide an inclusive overview of microbial sources of laccases and also sums up the role of microbial laccases in the valorization of LCB for efficient bioethanol production.

## 2 Lignocellulosic biomass conversion

Lignocellulosic biomass can produce maximum sugar amounts through fermentation, ultimately mounting the production of biofuel (E4tech Re-Cord, and Wur, 2015; Fillat et al., 2017). Conversion of LCB involves the processes of pretreatment and hydrolysis however, bioethanol production is chiefly dependent on the hydrolysis step (Binod et al., 2011). Yet, for efficient hydrolysis, pretreatment is regarded as a crucial step to alter lignocellulosic structure (Fillat et al., 2017).

### 2.1 Pretreatment

**FIGURE 2:**
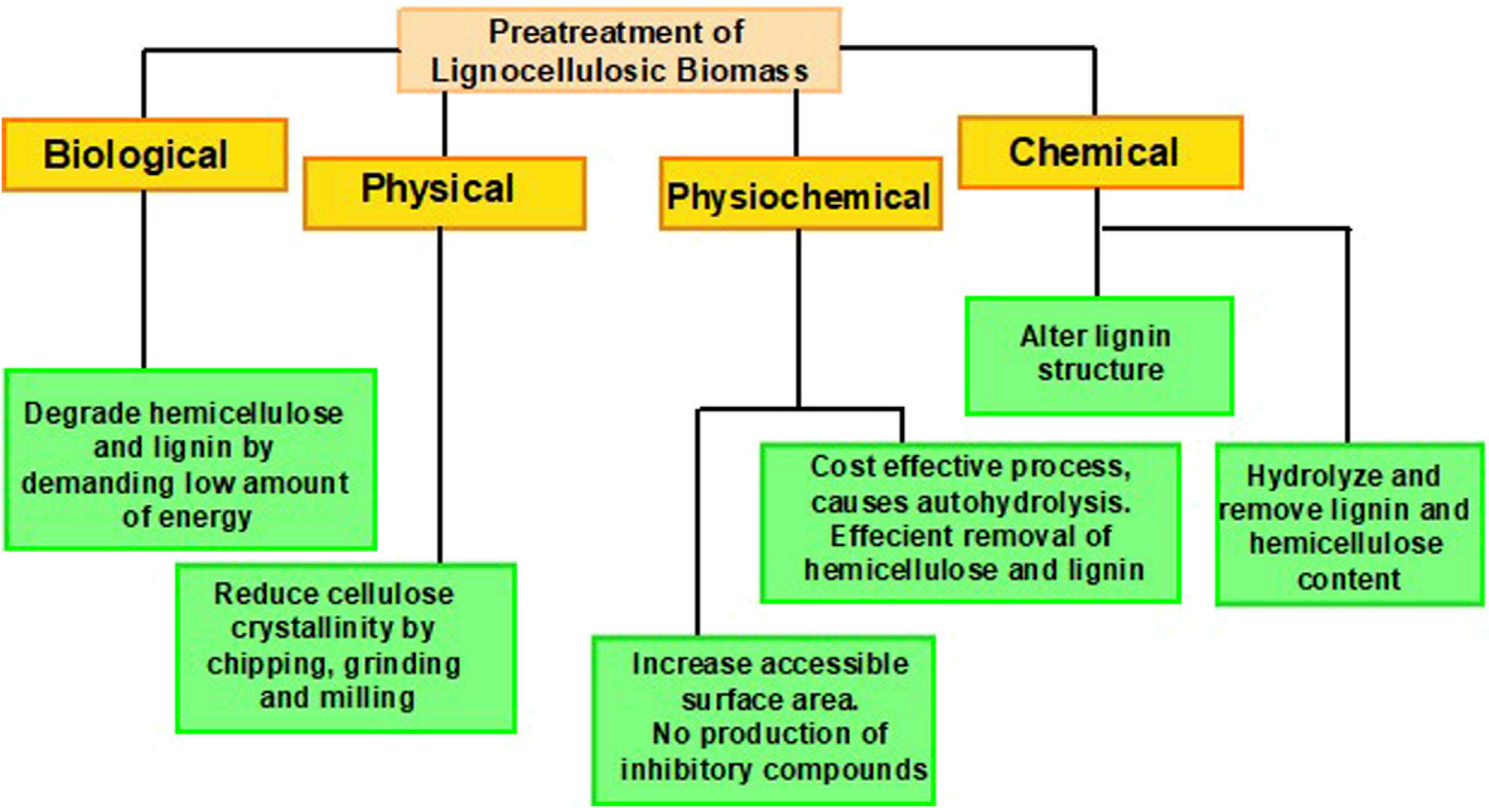
Amelioration in pretreatment technology.

Pretreatment of LCB is an imperative procedure to make lignin, cellulose, and hemicellulose more pertinent for enzymatic action by increasing amorphous regions. These products are the core building blocks of lignocellulosic compounds, which makes them defiant to the action of hydrolytic enzymes (Santos et al., 2015). Utilization of LCB for its biochemical conversion into biorefineries necessitates pretreatment to disrupt the inter-component associations between these constituents in the cell wall (Kumari and Singh, 2018). The main objectives of pretreatment comprise; creating a disturbance in the crystalline arrangement of cellulose, attaining efficient hemicellulose elimination and lignin degradation, enhancing the permeability of pretreated constituents, declining the degree of polymerization, and bioethanol production with bare minimum cost (Ji et al., 2017; Espirito Santo et al., 2018; Solarte-Toro et al., 2019). To make the process profitable, pretreatment must help escalate the effectiveness of hydrolysis and fermentation by diminishing the inhibitors besides raising the sugar formation (Chiaramonti et al., 2012). Several advancements have been carried out regarding the pretreatment of LCB, as illustrated in Figure 2.

### 2.2 Hydrolysis of LCB

Hydrolysis is performed to depolymerize lignocellulosic biomass into simpler sugars, and the degree of hydrolysis relies on the structural arrangements of lignocellulosic compounds. In comparison to hemicellulose, hydrolysis of cellulose is quite tricky due to its crystalline arrangements and the presence of β-D1, 4-glycosidic linkages. This indestructible structural organization makes cellulose exceedingly dense and hard to rupture. Physical (mechanical processes, milling, extrusion, microwave radiation, and extrusion), physicochemical (steam explosion, liquid hot water, ammonium fibre, and CO_2_ explosion, and wet oxidation), and chemical (alkaline, acid, ionic liquid, organo-solvent, technologies, deep eutectic solvent) pretreatment technologies effectively break down tough biomass for further processing, but they have several limitations. For instance, physical pretreatment methods are energy and cost-intensive as they operate by creating variations in pressure and temperature simultaneously. Thus, requiring high power, energy consumption, and increased production costs. While, chemical pretreatment generates harmful effluents and inhibitors, ultimately causing environmental contamination (Chami Khazraji and Robert, 2013; Abo-State et al., 2014; Shukla et al., 2023). For chemical hydrolysis, dilute, and strong acids are usually utilized. Hydrolysis with dilute acid requires 1%–3% sulfuric acid concentration at a temperature ranging from 180°C to 240°C. During this process, there also appears the formation of several inhibitors, succeeding in the production of hydrolysate and generating little sugar yield. However, the problem can be resolved by setting two temperature ranges such as 140°C–160°C and 160°C–180°C for hemicellulose solubilization and cellulose transformation, respectively, which upsurges the product recovery rate up to 20%. Hydrolysis with strong acid necessitates 20%–40% acid concentration with temperature maintained at 50°C–100°C, leading towards maximum yield, i.e., up to 90% (Hamelinck et al., 2005). Although chemical hydrolysis exhibits several positive points yet, it is unappealing and non-competitive due to the production of inhibitors (Abo et al., 2019).

Enzymatic hydrolysis is a type of hydrolytic reaction by which sugars are set free after the pretreatment of LCB. Ligninases, cellulases, and hemicellulases are the enzymes that usually catalyze this reaction (Rosdee et al., 2020). It is an eco-friendly process and is the foremost step for bioethanol production with minimal energy charge. This process offers a relatively lower generation of acid waste, inhibitory compounds, and other unwanted products. Furthermore, it also eliminates the requirement for corrosion-free equipment for processing (Moreno et al., 2017; Amit et al., 2018). Despite all these positive aspects, some limitations are also associated with enzymatic hydrolysis, such as lignin residues left in pretreated materials limit the process by inhibiting the availability of sugars for the action of hydrolytic enzymes or vice versa, ultimately diminishing the saccharification yield. Various enzymes or substrate-related factors in both positive and negative aspects affect enzymatic hydrolysis. However, enzymatic hydrolysis is still preferred over chemical one because of the non-toxic and biodegradable nature of the enzymes (Pino et al., 2018). To augment the efficiency of enzymatic hydrolysis, the copper-containing oxidases family, i.e., laccases are being used extensively for biofuel (particularly bioethanol) production. Laccases have vast practices in many industrially crucial processes and other biotechnological fields into the bargain. Moreover, laccases can oxidize phenolic, non-phenolic, toxic, and non-toxic substrates, further raising their global value (Hilgers et al., 2018). The potential of microbial lacasses in pretreating LCB residues for bioethanol production has been well studied in the literature. Lacasses degrade the complex polyphenol structure that constitutes lignin, which is the chief recalcitrant component in lignocellulosic structure. Lacasses being extracellular, inducible, and less specific are widely utilized for biomass pretreatment for biofuel production (Plácido and Capareda, 2015).

## 3 Role of microbial laccases in valorization of lignocellulosic biomass to bioethanol

During pretreatment of LCB, some inhibitors such as phenolic compounds, furan derivatives, levulinic acid, aromatic and inorganic compounds, aliphatic acids, formic acids, and extractives are produced as by-products which create hindrance during the hydrolysis (Maulana Hidayatullah et al., 2020; Qiao et al., 2021). Furthermore, lignin residues in pretreated lignocellulosic materials compress the concentration of hydrolytic enzymes by adsorbing them non-specifically, eventually constraining the saccharification process (Berlin et al., 2006). Laccase-mediated detoxification and delignification processes are generally carried out to decrease the inhibitor concentration and eliminate lignin compounds. These processes require no additional chemicals and occur with minimum energy consumption under milder conditions (Moreno et al., 2015). They also augment the accessible surface area and play a crucial role in reducing the ineffective joining of hydrolytic enzymes, eventually enhancing the worth of both processes (Fillat et al., 2017).

### 3.1 Why microbial laccases?

As described earlier, laccases are nonspecific and versatile enzymes that are involved in a wide range of processes, including lignin degradation, biofuel synthesis, and biorefinery of biomasses. Besides biofuels production, lacasses are widely utilized for the synthesis of pigments and are also known for their role in the production of antibiotic compounds. They are also extensively studied for their applications in the production of feed, food, and several other consumer products. Furthermore, they are involved in detoxification of toxic compounds and also have potential applications in bioremediation and wastewater treatment (Tiwari et al., 2023). Lacasses also play a vital role in generating bio-based chemicals such as vanillin, catechol, and syringol (Kim, 2021). Moreover, laccase can facilitate the oxidation of lignin compounds, which makes it easier to extract and utilize the sugar molecules and lignocellulosic material components for the production of bio-based plastics (Farmer et al., 2018).

Over the past, fossil fuels were widely utilized as an energy source, both in the industrial and transportation sectors. The increase in energy demand and depletion of fossil fuels augmented the advent of biofuels in the 19th century. In the mid-1970s, biofuel gained much attention in several countries, including Brazil and the United States, due to their fewer toxic effects and more economic gains over fossil fuels. Since then, the utilization of biofuels has been upgraded to 52,219 Ktoe (Kilotonnes of oil equivalent) from 8,082 thousand tonnes (Importance, 2014). Laccases are widely used for bioethanol production (A., 2013) and turned out to be the most efficient tool for valorizing LCB beyond its vast applications in other industrial sectors. However, the cost of these enzymes makes them unbefitting for implementation on a commercial scale. Concisely, laccases have the potential to be an eco-friendly tool for pretreatment of lignocellulosic materials’ delignification and detoxification, enhancing the fermentation and saccharification processes in biorefineries. Literature also depicts that *Trichoderma asperellum* strain BPLMBT1’s thermostable laccase efficiently delignified LCB, facilitating the synthesis of biohydrogen from delignified biomass (Shanmugam et al., 2018).

**FIGURE 3:**
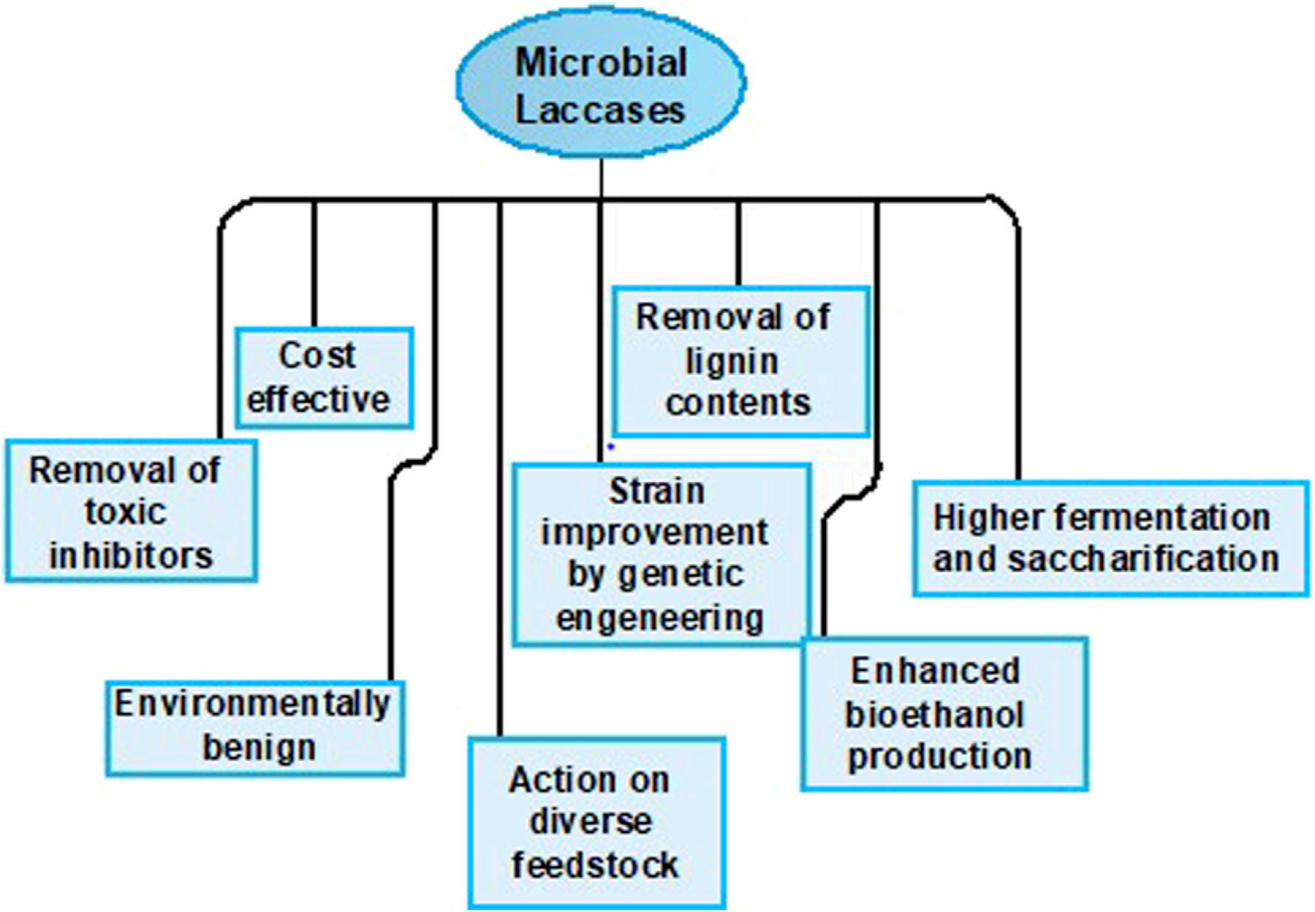
Potential attributes of microbial laccases.

For this reason, interest has ascended in the production of laccases by microbial sources to reduce the process expenses. Microbial laccases are competent catalysts and exhibit high stability. They possess broad specificity for substrate and can be easily cloned and expressed in the host. Furthermore, they can oxidize both non-toxic and toxic substrates and also acquire higher redox potential, providing economical services for industrial purposes as presented in Figure 3 (Shraddha et al., 2011; Singh et al., 2011; Prins et al., 2015; Mate and Alcalde, 2017).

### 3.2 Microbial sources of laccases

Yoshida initially described laccases, identified from a tree *named Rhus vernicifera* (Wang Z. et al., 2018). Presently, laccases are recognized and studied chiefly in fungi, but these enzymes are also present in some bacterial species as well. White rot fungi are major producers of extracellular laccases throughout their secondary metabolism (Rodríguez-Couto, 2017; Singh and Gupta, 2020). Laccases have been purified from several strains of fungi (Guimarães et al., 2016), but the *Trametes* genus is typically studied (Wang Q. et al., 2018). Furthermore, laccases produced from basidiomycetes and ascomycetes possess high redox potential, which makes these fungal groups most significant (Wu et al., 2019). Studies have also indicated the active production of laccases from *Coprinus comatus*, *Lentinus edodes*, and *Lepista luscina* sp. Unlike fungal species, laccases purified from bacterial strains have some exceptional characteristics such as bacterial-derived laccases are either extracellular or intracellular and are usually stable even at high pH and temperature (30°C–80°C) ranges, making them a potential candidate for laccases production (Chauhan et al., 2017). Among bacterial species, *Streptomyces*, *Azospirillum*, *Rhodococcus*, *Geobacillus*, *Staphylococcus*, *Lysinibacillus sphaericus*, *Bacillus* spp., *Aquisalibacillus*, *Pseudomonas*, *Delftia*, *Alteromonas*, *and Enterobacter* are some main sources (Axelsson et al., 2012; Muthukumarasamy et al., 2015; Neifar et al., 2016; Rezaei et al., 2017). Moreover, some yeast species naturally produce laccases, including *Kluyveromyces lactis*, *Pichia methalonica*, and *Pichia pastoris*, etc. Some yeast species are also being utilized for recombinant laccases production and basidiomycetes yeast are the most important among all. Some chief microbial sources of laccases are given in Table 1.

**TABLE 1:** Bacterial, fungal and yeast sources of laccases.

*Microbial sources*	References
Fungal sources	
*Phanerochaete chrysosporium*	Linares and Loske (2018), Ghobadi Nejad et al. (2019)
*Lenzites*, *betulina*	Jasim, 2017
*Trametes versicolour*	Pazarlioǧlu et al. (2005), Pinheiro et al. (2020), Tapia-Tussell et al. (2020)
*Pleurotus ostreatus*	de Freitas et al. (2017), Mendonc et al. (2018), Song et al. (2020)
*Theiophora terrestris*	Shraddha et al. (2011), Dana et al. (2017)
*Phlebia radiate*	Muthupriya et al. (2019)
*Trichoderma atroviride*	Chakroun et al. (2010), Umar (2021)
*Monocillium indicum*	Irfan et al. (2018), Majolagbe et al. (2020)
*Pycnoporus sanguineus*	Salcedo Martnez et al. (2013)
*Trichoderma harzianum*	Ranimol et al. (2018)
*Pycnoporus cinnabarinus*	Henske et al. (2018), Preethi et al. (2020)
*Gaeumannomyces graminis*	Yang et al. (2017)
*Ophiostoma novo-ulmi*	Nicolcioiu et al. (2018)
*Coriolus hirsutus*	Karp et al. (2013), Sun et al. (2013)
*Laetiporus sulphureus*	Shraddha et al. (2011)
*Coprinus comatus*	Bertrand et al. (2013)
Bacterial sources	
*S. lavendulae*	Mishra et al. (2017)
*Marinomonas mediterranea*	Jimenez-Juarez et al. (2005)
*Stenotrophomonas maltophilia*	Galai et al. (2009)
*Streptomyces coelicolor*	Lee et al. (2019)
*Lysinibacillus sphaericus*	Chantarasiri et al. (2017)
*Streptomyces cyaneus*	Arias et al. (2003)
*Pseudomonas putida*	Mandic et al. (2019)
*Bacillus subtilis*	Lee et al. (2019)
*Alteromonas* sp.	Maia et al. (2021)
Yeast sources	
*Saccharomyces cerevisiae*	Aza et al. (2021)
*Pichia methalonica*	Antošová and Sychrová (2016)
*Cryptococcus neoformans*	Zhu and Williamson (2004)
*Yarrowia lipolytica*	Antošová and Sychrová (2016)

Compared to other industrial processes, laccases production through fermentation is considered low to moderate in terms of energy consumption. Fermentation primarily uses energy for maintaining culture growth conditions like temperature and agitation. However, these requirements are generally less demanding than those needed for high-heat chemical processes. Even purification often utilizes chromatography techniques, which may involve low-pressure pumps and refrigeration. The sterile environment required for laccase production disrupts the natural microbial communities present in bioreactors and the specific growth medium and conditions used for laccase-producing organisms favor their growth over other microbes (Malhotra and Suman, 2021).

## 4 Laccases; structure, functions, and mechanism

Microbial laccases are a group of versatile enzymes with immense potential in various biotechnological applications, particularly those related to biomass conversion. Lacasses are superior to other enzymes such as peroxidases and cellulases because they have broader substrate specificity, oxidizing a wide range of phenolic and non-phenolic compounds using molecular oxygen (O_2_) as the final electron acceptor. While, peroxidases primarily target specific electron donors in the presence of H_2_O_2_ (Rodríguez-Couto, 2021). Laccase pretreatment can improve cellulose accessibility for cellulases, facilitating biofuel production or other applications. It can be better understood by discussing the structure of laccases.

**FIGURE 4:**
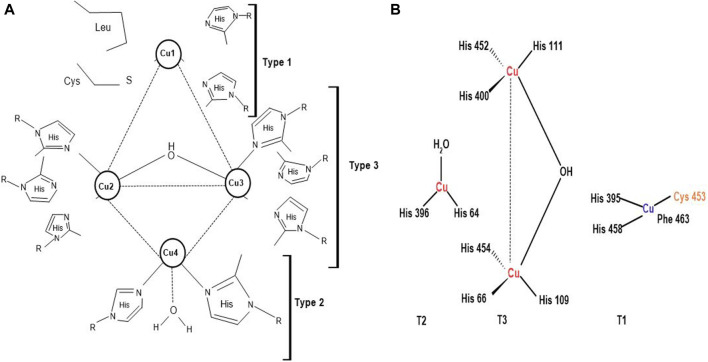
**(A)** Copper coordination network of laccases; **Type 1**: Blue copper, **Type 2**: Normal copper, and **Type 3**: Binuclear copper core (Agrawal et al., 2018) **(B)** Active site of laccases (Prajapati, 2018).

Laccases are usually dimeric or tetrameric glycoproteins, exhibiting 500 amino acids organized into three domains, having beta-barrel topology. They contain four copper atoms per monomer at their active sites, which partake in water production by reducing oxygen molecules. Laccases are classified into three main groups; blue copper (type-1 copper), normal copper (type-2 copper) and binuclear copper cores (type-3 copper) (Figure 4) (Plácido and Capareda, 2015; Yang et al., 2017). Every four atoms of copper have their particular attribute towards electron spin resonance signals. Type-1 copper is blue copper due to its intense absorption close to 600 nm, ensuing blue color. Blue copper has ligand collaboration among one methionine plus two histidine and cysteine molecules and exhibits high redox potential and capacity to oxidize the substrate. Some laccases show lesser absorption, i.e., near 600 nm, and are termed white and yellow laccases depending on the oxidation state of the Zn, Cu, and Fe atoms they contain (Radveikienė et al., 2021; Sondhi et al., 2021). Type-2 or normal copper exhibits zero absorption in the visible spectrum range (Wang S. N. et al., 2018) and uses water and 2 histidine molecules as a ligand (Babiker and Hamadttu, 2019). Type-3 copper uses one hydroxyl bridge and three histidine molecules as ligands, displaying absorption at 330 nm. Laccases have also been categorized into high and low redox potential laccases, notably fungal and bacterial laccases, respectively (Yin et al., 2019; Janusz et al., 2020) on account of the properties and structures of laccases copper cores and ligands along with the distance between the copper atoms (Rencoret et al., 2008). This unique structural arrangement of laccases helps in toxin and lignin degradation of LCB, validating worth functioning of the enzyme (Bagewadi et al., 2017; Janusz et al., 2020).

### 4.1 Mechanism of action of laccases

Oxidation catalyzed by laccases can be direct, indirect, or mediator-associated depending upon the substrate size and redox potential. During direct oxidation at Type-1 copper atom, unsettled reactive radicals are generated by one electron substrate oxidation. After four consecutive cycles, the electrons become capable of producing two water molecules by reducing one molecule of oxygen (Kudanga et al., 2011). For mediator-associated or indirect oxidation, the laccase mediator system is used. LMS oxidizes small substrates with little redox potential at first and then uses its generated radicals for oxidizing complex substrates with relatively higher redox potential, which makes it imperative for biofuel manufacturing (Kudanga and Le Roes-Hill, 2014).

**FIGURE 5:**
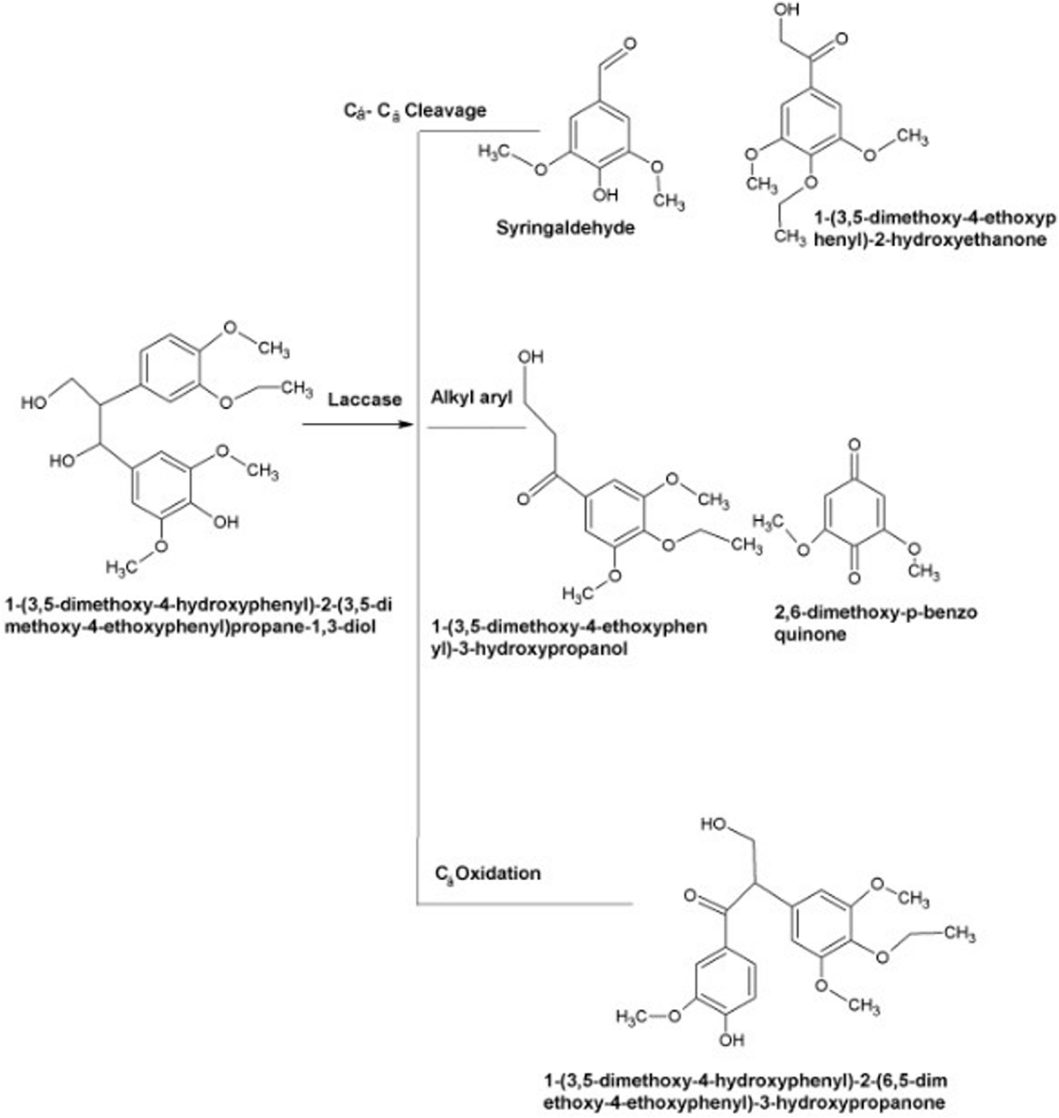
Mechanism of lignin compound (agro-industrial waste) oxidation via laccases as a substitute to acquire fermentable carbohydrates for fuel making (Wei and Xianghua, 2009; Sanchez et al., 2011).

As described earlier, laccases produce reactive radicals by substrate oxidation resulting in the generation of water molecules by reducing oxygen with the engagement of copper atoms present at their catalytic center. These copper atoms are also involved in the degradation of lignin. In the very first step, blue copper oxidizes lignin. Later on, Type-1 copper transfers electrons to Type-2, which further transfers these electron to Type-3 (Bishwakarma et al., 2016). Substrates including aromatic diamines, polyphenols, and methoxy-substituted phenols are generally oxidized by laccases via Cα oxidation, Cα-Cβ cleavage and alkyl-aryl cleavage. The cofactor of laccase is oxygen rather than hydrogen per oxidase, necessitating its role during the delignification process. In some cases, laccases require mediators for the aforesaid process due to its vast magnitude and complex lignin structure (Singh Arora and Kumar Sharma, 2010; Sanchez et al., 2011). The mechanism of laccases action is illustrated in Figure 5.

## 5 Laccase practices for detoxification of pretreated material

Laccases are extensively used to minimize the toxicity of various pretreated compounds and are exceptionally helpful in the detoxification of LCB, eventually resulting in increased efficiency of microbial fermentation (Jurado et al., 2009; Moreno et al., 2012, 2013a; Guo et al., 2013). The pH is a significant factor for the efficient functioning of laccases as slight variations in pH lead to structural alterations in enzymes, consequently making them inactive. These enzymes produce phenoxy radicals by oxidizing phenolic compounds (Sun et al., 2021) and by interacting with pretreated lignocellulosic material via polymerization, yielding oligomers with low inhibitory power (Parawira and Tekere, 2011). Studies have shown that laccases reduce the incidence of pre-hydrolysate phenolic compounds from 88% to 92% (Moreno et al., 2012).

### 5.1 Microbial laccases mediated detoxification

Laccases being oxidative enzymes, cleave the carbon-carbon and ether bonds in lignin during the formation of phenoxy radicals (De La Torre et al., 2017). Kolb et al. (2012) described the catalytic activities of laccases produced from *Trametes versicolor* while working on inhibitory compounds. It was proposed that in laccase presence, the reaction time of 1 h for ferulic acid, syringaldehyde, and p-coumaric, 24 h for vanillin, and more than 1 week for 4-hydroxybenzaldehyde is required for their complete removal. Studies have reported 76% and 92%–95% phenol reduction by treating pretreated LCB with laccases extracted from *Coltricia perennis* (Martínez et al., 2009; Kalyani et al., 2012) and *C*. *rigida* or *Pycnoporus cinnabarinus*, respectively (de Abreu Domingos and da Fonseca, 2018). Two other studies have also demonstrated similar results for the reduction of phenolic compounds in pretreated biomass by treating them with *P. cinnabarinus*, *C. rigida*, *T. versicolor* and *Thapsia villosa* derived laccases (Kalyani et al., 2012; Moreno et al., 2012).

Laccase activity for phenolic compounds can be enhanced by electron-releasing groups, which lessen the electrochemical potential of relative phenols. Moreover, the presence of methoxy and ethylene groups is also concerned with the escalation of laccases affinity for phenolic compounds (Camarero et al., 2005; 2008). Fungal laccases are more active toward phenols than bacterial laccases due to relatively high redox potential (Glazunova et al., 2018).

### 5.2 Detoxification through microbial laccases and its effect on enzymatic saccharification

The biofuel production from laccases pretreated lignocellulosic biomass is becoming a great concern internationally (Guo et al., 2018). Thereby, processes of laccase-mediated detoxification have also been assessed concerning enzymatic saccharification owing to their ability to enhance the saccharification yield. Following this concept, Kalyani et al., (2012), showed that the laccases from *C*. *perennis* increase the saccharification output by up to 40% by decreasing the phenolic contents in pretreated biomass. Contrastingly, there are some phenolic laccase derivative complexes produced during the saccharification process that decrease the saccharification yield. Several studies highlighted a drop in the sugar concentration when laccases from *C. rigida*, *P. cinnabarinus* and *T. villosa* were used for the pretreatment of LCB, followed by enzymatic hydrolysis (Tabka et al., 2006; Jurado et al., 2009; Moreno et al., 2012, 2013a). De la Torre et al. (2017) also supported this concept by elucidating poor saccharification yield from pretreated LCB. Oliva-Taravilla et al. (2015b, 2016) observed that oxidation of phenolic laccase derivative compounds, i.e., syringaldehyde and vanillin, through laccases of *Myceliophthora thermophile*, led to the formation of the oligomeric product, which decreases the saccharification yield to 32.6% and 46.6%, respectively. In the same study, more than a 50% decrease in the activity of β-glucosidase and cellulase was observed due to the presence of vanillin. Subsequently, this increased competition between laccases and hydrolytic enzymes eventually lessened glucose recovery (Oliva-Taravilla et al., 2015a). Hence, to overcome this issue, the laccase mediator system has been developed as it is a proficient mean to upturn the saccharification yield (Giacobbe et al., 2018) by abating undefined enzymatic bindings, ultimately improving the efficacy of enzymatic hydrolysis (Heap et al., 2014).

### 5.3 Detoxification through microbial laccases and its effect on fermentation yield

Yeast, particularly *S. cerevisiae* is the most common microorganism used for bioethanol production besides estimating laccase detoxification effects. Several studies have demonstrated the enhanced output of bioethanol under laccase activity such as pretreated LCB when detoxified by laccases (produced from *T. versicolor* or *C. rigida*) favored more sugar consumption for *S. cerevisiae* growth which ultimately improved the bioethanol yield (Antošová and Sychrová, 2016). Similarly, laccases produced from *P. cinnabarinus*, when employed, induced high cell viability, reduced lag phase, and elevated the rates of bioethanol production (Moreno et al., 2013b; 2016a). Fang et al. (2015) also described the use of laccases extracted from *Ganoderma lucidum* for detoxifying pre-hydrolysate of steam-exploded LCB, which also resulted in improved yeast growth. Laccases from *Cyathus stercoreus* detoxified 77.5% phenolic compounds, and *T. versicolor*-derived laccases offered 80% detoxification of LCB with bioethanol yield of 0.374 and 0.43, respectively (Plácido and Capareda, 2015).

Research efforts demonstrated that fungal laccases, mainly derived from *Aspergillus oryzae*, also raise the fermentation efficiency to 6.8% (Li et al., 2014). Moreover, other fermenting yeasts also confer the same laccase detoxification effects as *S. cerevisiae*. Chandel et al. (2007) demonstrated that sugarcane bagasse prepared with laccases obtained from *C. stercoreus* fostered efficient working of *Candida shehatae* during acid hydrolysate fermentation. Furthermore, the presence of thermo-tolerant yeast elevates the yield and efficiency of fermentation besides saccharification processes, fortifying the concept that temperature is an imperative parameter that has a direct influence on fermentation. *Kluyveromyces marxianus* is a thermotolerant yeast and can withstand >40°C temperature (Abdel-Banat et al., 2010). Fermentation of wheat straw with *Kluyveromyces marxianus*, succeeded by steam explosion and laccase (from *P. cinnabarinus*) assisted detoxification provided ethanol yield similar to the one attained from *S. cerevisiae* (Moreno et al., 2013b). A higher concentration of bioethanol is also achieved by setting off fermentation and saccharification practices at high-substrate uniformities, in consort with laccase detoxification (Rosgaard et al., 2007). In the case of pretreated wheat straw with 12% (w/v) substrate loading, detoxification by laccases derived from *P. cinnabarinus* resulted in 16.7 g/L bioethanol concentration by *K. marxianus* during solid substrate fermentation. Contrastingly, in the same study, 22 g/L of bioethanol concentration was achieved by utilizing laccases obtained from *P. cinnabarinus* when substrate loading was about 20% (w/v). At this uniformity, the F12 strain of *S. cerevisiae*, which consumes developed xyloses remained incapable of propagating, and this inhibition was controlled by microbial laccases particularly purified from *P. cinnabarinus* (Moreno et al., 2013d). *P. cinnabarinus*-derived laccases were capable of generating 32 g/L bioethanol production from pretreated LCB even at 16% (w/v) substrate loading (Moreno et al., 2013c).

## 6 Laccase practices for delignification of pretreated material

Laccases mediated delignification is an efficacious process to escalate the valorization of LCB to bioethanol by employing a laccase-mediator system or immobilized laccases (Malhotra and Suman, 2021). During this process, aromatic lignin radicals are created by the oxidation of lignin compounds which successively induce lignin delignification by splitting the aromatic ring, accompanying carbon-carbon or ether bond degradation (Ramalingam et al., 2017). This procedure requires specific cultural conditions, which, in detail, are discussed in Table 2.

**TABLE 2:** Cultural settings for laccases production and subsequent results.

Laccase source	Cultural settings			Substrate	Results	References
	Technique	Temperature °C	Time			
*Kluyveromyces marxianus* sp.	SSF^a^	40	15 days	Wheat Straw	Detoxification of LCB	Moreno et al. (2016b)
*Trametes versicolor* sp.	SmF^b^	25	30 days	Corn Straw	Increases oxidative treatment efficiency	Yu et al. (2010b)
*Lentinus tigrinus* sp.	SSF	28	7 days	Wheat Straw	LCB degradation	Salvachúa et al. (2011)
*Pleurotus*ostreatus sp.	SSF	30	5 days	Sugarcane bagasse	Delignification of lignin contents of biomass	Karp et al. (2015)
*Pycnoporus cinnabarinus*	SSF	30	15 days	Lantana camara	20% increase in sugar yield	Gupta et al. (2011)
*Candida shehatae*	SmF	30	1 day	Sugarcane bagasse	Detoxify inhibitors and increase ethanol yield	Chandel et al. (2007)
*Cotylidia pannosa*	SmF	31	2.9 days	Wheat bran	Maximum laccase activity	Sharma et al. (2016)
*Stereum hirsutum*	SSF	25	21–49 days	Radiata pine	Increase surface porosity of biomass and lignin degradation	Shirkavand et al. (2017)
*Irpex lacteus*	SSF	28	15 days	Corn stalk	80% reduction in lignin contents	Yu et al. (2010a)
*Myceliophthora thermophila*	_	23	10 days	Wheat straw	Oxidize and chemically alter lignin	Kaparaju and Felby (2010)
*Thapsia villosa*	_	28	2 h	Wheat straw	Reduce toxicity of phenolic inhibitors and improve hydrolysate ferment ability	Jurado et al. (2009)
*Ureibacillus thermosphaericus*	_	50	24 days	Waste house wood	Remove toxic compounds along with reducing sugar loss	Okuda et al. (2008)
*Cerrena unicolor*	_	45	1–3 days	Steam exploded spruce	Amendments in lignin	Moilanen et al. (2011)
Basidiomycete *Euc*-1	SSF	28	35 days	Wheat Straw	Delignification resulting in LiP, MnP, and laccases production	Dias et al. (2010)

^a^ SSF, solid state fermentation.

^b^ SmF, submerged fermentation.

### 6.1 Delignification through laccases and bioethanol productivity

**FIGURE 6:**
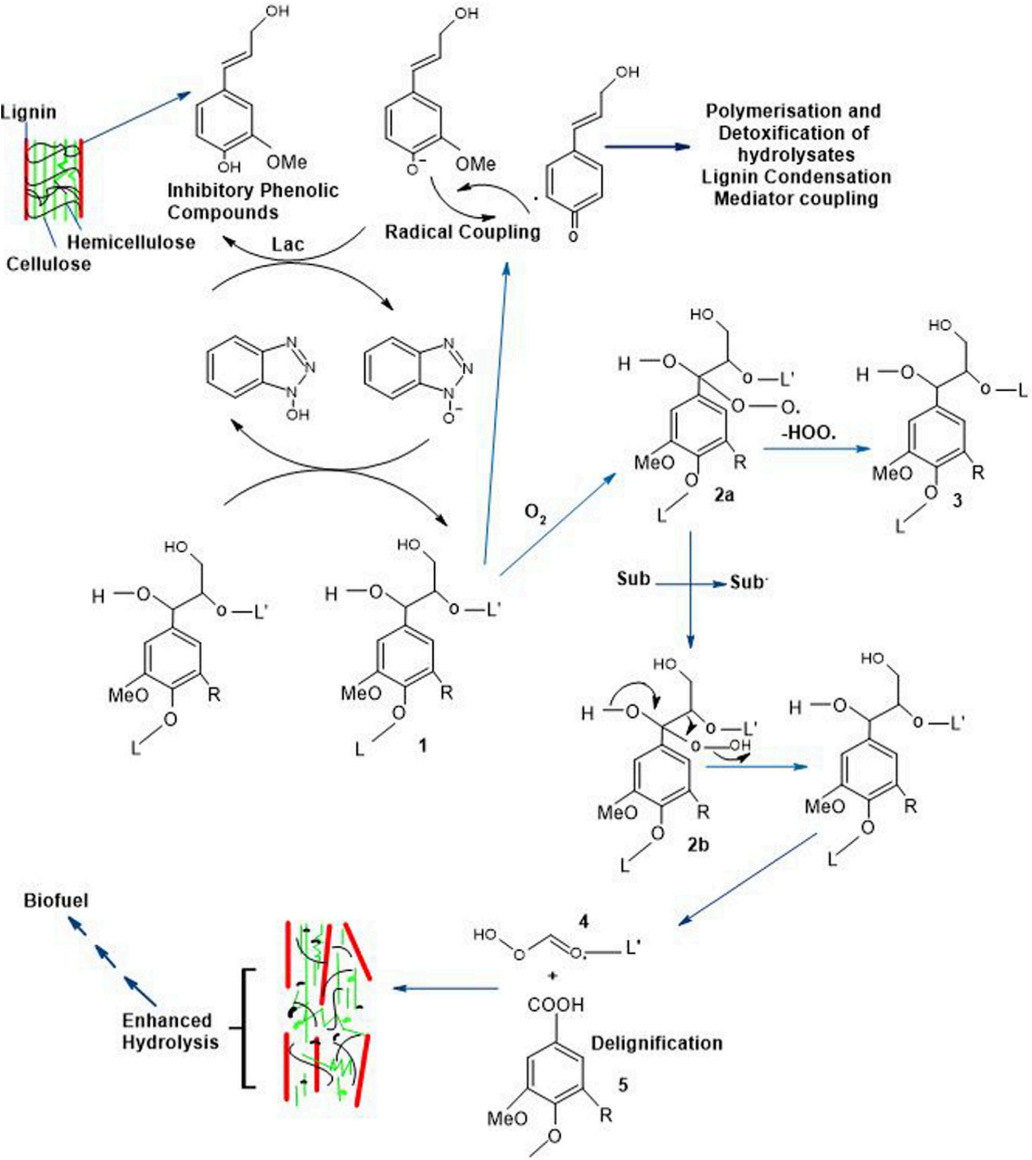
Delignification Strategies of Lignocellulosic Structure and Successive Tread in Bioethanol Production (R = H; Guaiacyl units, R = OMe; Syringyl units, L = Lignin, Sub = Substrate, Sub* = Radical and Lac = Laccases) (Du et al., 2013; Kudanga and Le Roes-Hill, 2014).

The accumulation and variety of inhibitors’ production hinge on the reaction conditions, biomass composition, and pretreatment methodologies (Bhatia et al., 2020; Agrawal et al., 2021). During biomass pretreatment, several phenolic inhibitors are generated from lignin. Laccases-mediated delignification assists in the removal of these inhibitory compounds for efficient production of bioethanol with increased yield, the mechanism of which is provided in Figure 6. The 1st step performed is the pretreatment which has increased the hydrolysis yield by successive hydrolysis step (2nd step) to form organic compounds that exhibit solubility in their nature (such as amino acids) by crashing down the insoluble organic compounds together with greater molecular weight compounds (i.e., lignin) in the presence of actinomycetes. In the 3rd phase, hydrolysis products are converted into alcohols besides hydrogen which leads the way to the production of biogas and methane in the 4th phase by conversion of carbon compounds, methanol and hydrogen by microbial action, particularly of methanococcus as well as methanobacterium genera (Bagewadi et al., 2017).

Numerous studies screen the delignification of pretreated biomass by laccases alone, prompting a boost in bioethanol production. Two such studies highlighted the attainment of 86% and 84%–89% delignification range by utilization of *Pleurotus*-derived laccases for *Ricinus communis* in addition to *Lantana camara* and *Bambusa bambos* materials, respectively, increasing the sugar yield up to 2.68 folds (Fillat et al., 2017; Kumar and Banerjee, 2018; Gujjala et al., 2019). It is also evidenced that supplementary sugar yield favors more bioethanol production both commercially and at a laboratory scale. Sitarz et al. (2013), stated that delignification of pretreated LCB from *G. lucidum* laccases dilated sugar yields by 75%, hence enhancing the bioethanol production. Rencoret et al. (2016) also claimed that laccases from *P. cinnabarinus* caused 18% delignification of LCB after extraction of alkaline peroxide, sooner or later increasing the sugar yield to 24%–25%. Another research reported that *C. perennis*-derived laccases upturn the saccharification yield to 48% by eliminating 76% of lignin-derived inhibitors (Bagewadi et al., 2017). (Rico et al. (2014) also demonstrated 50% delignification of pretreated biomass by laccases produced from *M. thermophile*, consuming methyl syringate as a mediator and conclusively improved the bioethanol productivity (Moreno et al., 2020). Meanwhile, Qiu and Chen, (2012) depicted that laccases attained from *Sclerotium* are liable to deteriorate the carbohydrate-lignin complex of LCB by amplifying glucose production.

Besides lignin removal, laccases also play an essential role in bioethanol production through the valorization of LCB by changing various properties of hydrolyses. Laccases are accountable for upgrading enzymatic hydrolysis and structural alteration of microfibers (Rocha-Martín et al., 2018). Amendments in porosity, hydrophobicity, and surface area of LCB by laccases aid in reducing the non-specific binding of hydrolytic enzymes; for instance, laccases from *Trametes hirsuta* raised the surface area and porosity of several alkali-extracted LCB, proliferating the saccharification yield and bioethanol production (Li et al., 2012). Despite all these positive aspects, a few drawbacks are also associated with using laccases alone for delignification of LCB, such as these enzymes can only oxidize phenolic lignin while non-phenolic lignin usually left untreated. However, to get over this problem, the laccase mediator system is widely used for executing appreciable delignification (Abdel-Hamid et al., 2013).

## 7 Laccase mediator system

The capability of laccases to batter and deteriorate lignin by collaborating with laccase mediators is considered a prodigious prospective for lignin valorization (Wang X. et al., 2018). Delignification with the laccase mediator system (LMS) causes non-phenolic and phenolic lignin degradation by the subsequent mediator and enzyme-catalyzed oxidative processes (Christopher et al., 2014). Delignification concerning LMS is a tranquil process in which small mediator particles get oxidized by laccases and then penetrate the dense structure of lignocelluloses, causing delignification of the sample. The efficiency of delignification through LMS is dependent on the combinations and practices of mediators and laccases; for instance, 1-hydroxybenzotriazole (HBT) with *P. cinnabarinus* laccases (PcL) and methyl syringate (MeS) with *Myceliophthora thermophila* laccases (MtL) are the most efficient LMS for delignification (Du et al., 2013).

### 7.1 Mechanism of laccase mediator system

**FIGURE 7:**
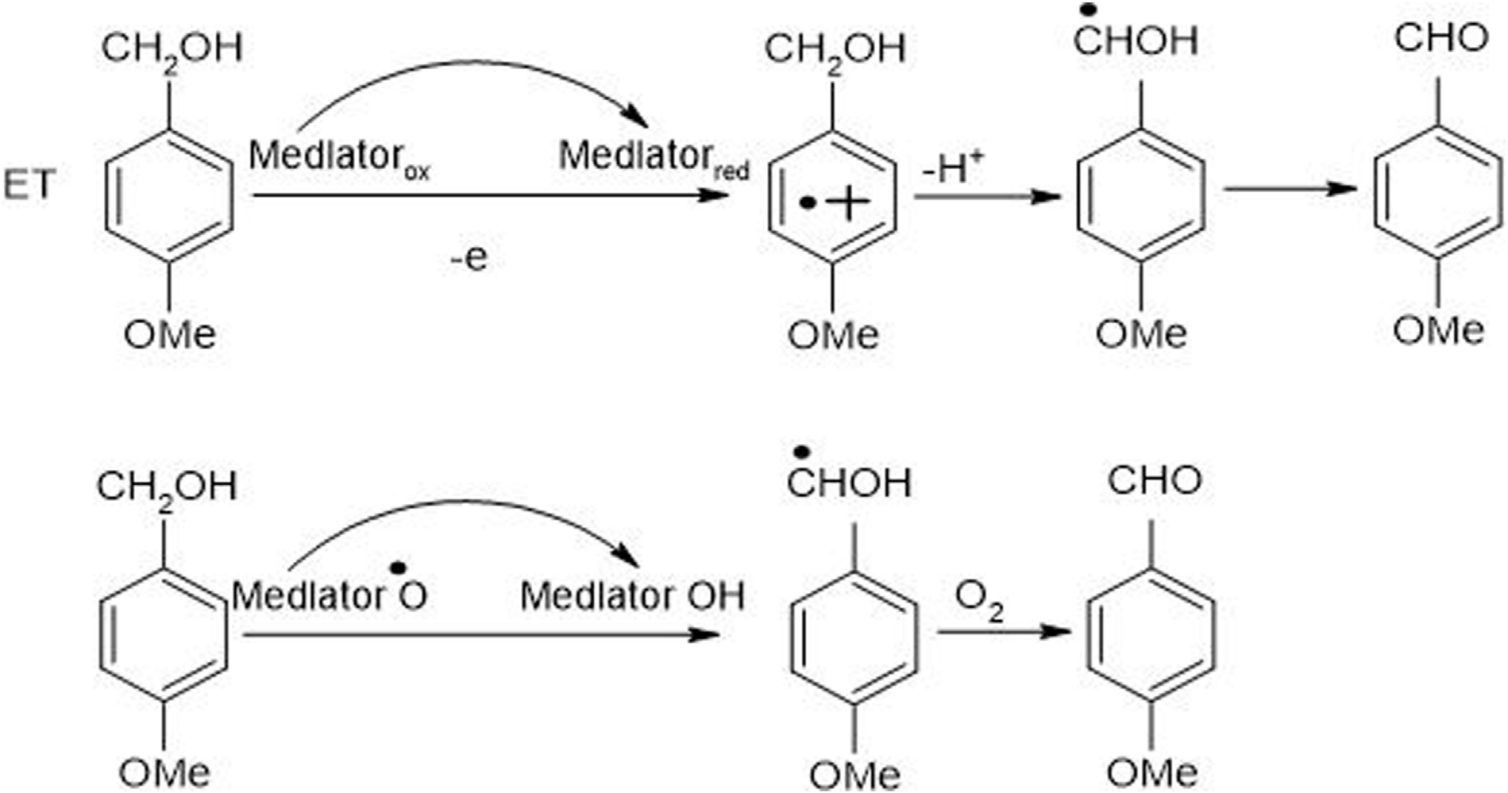
An anticipated mechanism for non-phenolic LMC (4-methoxybenzyl alcohol) oxidation, catalyzed by LMS, to 4-methoxy benzaldehyde along with electron and hydrogen atom transfer (Christopher et al., 2014).

The mechanism of LMS comprises oxidation of inhibitors particularly non-phenolic in nature by implementing three mechanisms such as electron transfer, ionic mechanism (Du et al., 2020) and radical hydrogen atom transfer (Figure 7). The indication for the existence of all these mechanisms is obtained by measuring the isotopic effects of intra-molecular kinetic and the pattern of degradation of non-phenolic lignin (Barreca et al., 2004).

**FIGURE 8:**
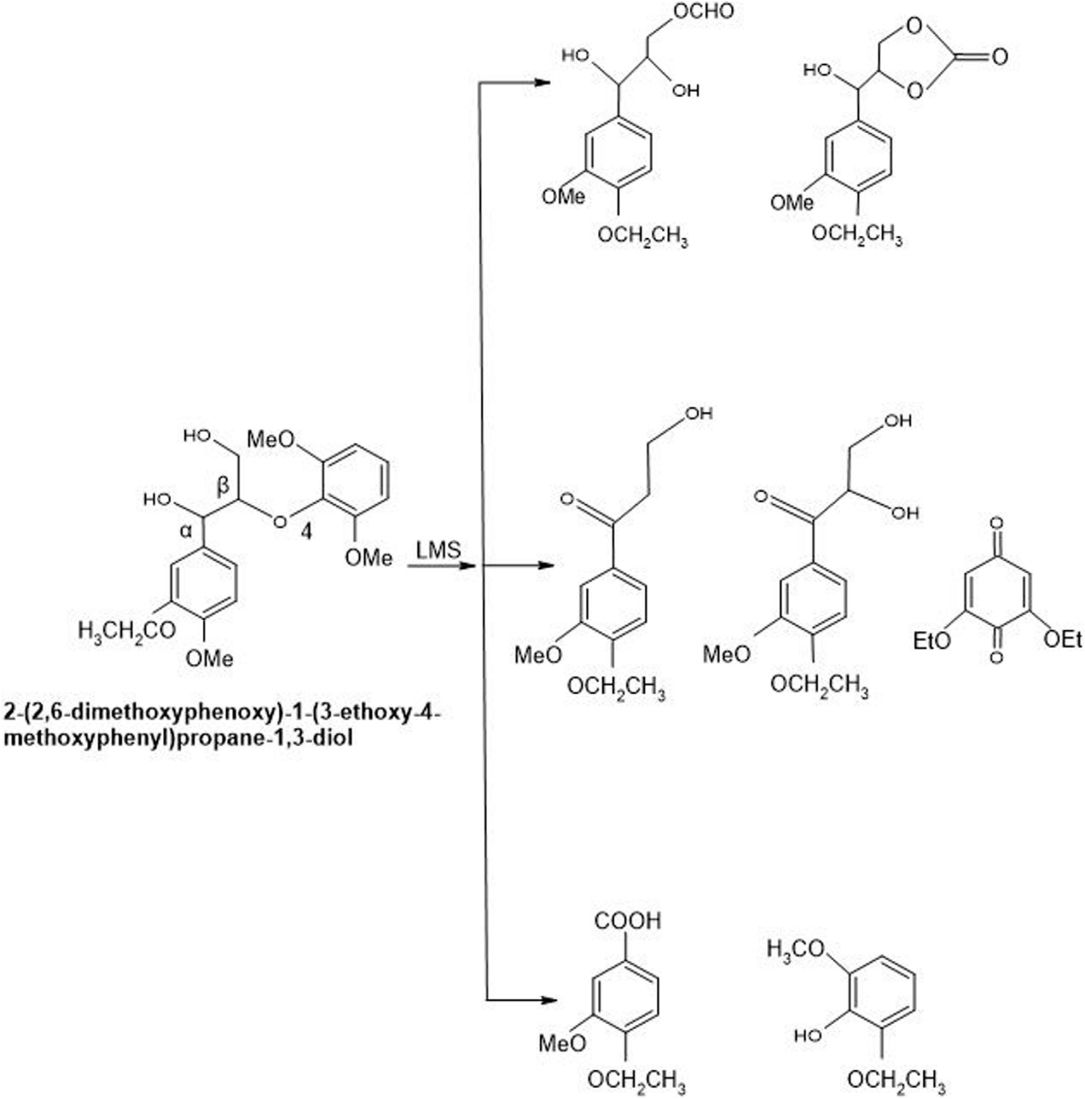
Non-phenolic compound (β-O-4 LMC) cleaved oxidatively by Laccase-HBT system of *T. versicolor*. By using laccase-HBT, the oxidation of β-O-4-linked non-phenolic lignin model compounds brought about Cα oxidation, aromatic ring-opening, and β-ether cleavage generation of carboxylic acids along with aromatic carbonyl compounds (Kawai et al., 2002).

Every mediator used in the laccase mediator system follows its exclusive oxidation mechanism. For instance, the presence of ABTS in LMS causes non-phenolic inhibitor oxidation through the electron transport route. On the contrary, laccase-TEMPO follows an ionic mechanism while laccase-HBT monitors a radical mechanism to oxidize inhibitors (Baiocco et al., 2003). The mechanism adopted by laccase-HBT is quite complex as a coupling intermediated product, i.e., β-aryl radical cation, as well as benzylic Cα radical, are formed during this course. It resulted in the formation of benzotriazole or some stable complexes having the ability to covalently bind some HBT to lignin. Laccase-HBT breaks down the C_α_‐C_ß_ link during oxidation and results in the formation of carboxylic acid assemblies in treated LCB (Figure 8). Occasionally, oxidation mechanism also affects the molecular weight of lignin model compounds. Few studies highlighted that during the oxidation of non-phenolic and phenolic lignin model compounds, the presence of laccase-HBT, particularly from *T. villosa* caused a 4 to 5 times decrease in their molecular weight, respectively. Some emblematic products are also being generated from the degradation of lignin by using LMS, including 2,6-dimethoxy-4-((E)-prop-1-enyl)benzaldehyde, 2,6-dimethoxy-4-methylbenzaldehyde, and 4-ethyl-2,6-dimethoxybenzaldehyde (Christopher et al., 2014).

### 7.2 LMS mediated delignification and enzymatic hydrolysis

Several research studies have demonstrated the efficiency of LMS in enhancing the processes of delignification (Table 3) and enzymatic hydrolysis such as Rico et al. (2014) studied that pretreatment using LMS led to the 50% delignification of LCB of *Eucalyptus globulus* wood by subsequent alkaline peroxide abstraction. Similarly, Zanirun et al. (2015) executed 8% delignification of oil palm LCB using HBT mediator in conjunction with *Pycnoporus sanguineus* laccases. The same study also reported 8.7% delignification of the sample when the ABTS mediator was being exercised, ultimately increasing fermentable sugar yield to 30 g/L.

**TABLE 3:** Laccase mediator system aptness for pretreated lignocellulosic feedstock delignification.

Pretreated contents	Laccase mediator system (LMS)	Practical effects	Exploits	References
Wheat straw is being pretreated with liquid warm water	Laccase *P. sanguineus* H275 with mediator VIO	Lignin is lost almost 97%	Increase in the production of sugar up to 19.98%	Lu et al. (2010)
Elephant grass pretreatment with ultrasound	Laccase from *T. hirsuta* having ABTS mediator	69% range of delignification	Enhanced availability of cellulose	Nagula and Pandit (2016), Scopel et al. (2020)
Spruce LCB	Laccases from *T. hirsute* with TEMPO	Oxidation of cellulose plus modification of lignin ensued in decrease in the uncreative cellulases adsorption	49% increase in enzymatic hydrolysis	Moilanen et al. (2014)
Eucalypt timber which is Steam-exploded	Laccase from *M. thermophila* along with HBT mediator	Increase in the production of secondary OH groups and degree of condensation after oxidizing lignin	Increase in the production of sugar to some extent	Martín-Sampedro et al. (2012), Kang et al. (2021)
Spruce LCB	Laccases from *T. hirsute* with ABTS.	Modification of lignin ensued in decrease in the uncreative cellulases adsorption	Rise in enzymatic hydrolysis up to 54%	Moilanen et al. (2014)
Oil palm LCB	*P. sanguineus* laccases in combination with HBT mediator	8% delignification	Increase in sugar yield	Zanirun et al. (2015)
Wheat straw which is acid steam-exploded	Laccase from *T. versicolor* with mediator HBT monitored by alkaline peroxide extraction	Oxidation of lignin which is discovered by Py/GC-MS TMAH	Almost 2.3 g/L increase in glucose discharge	Heap (2014)
Pretreated Oil palm LCB	*P. sanguineus* laccases grouped with ABTS mediator	8.7% delignification	30 g/L increase in fermentable sugar yield	Zanirun et al. (2015)
Pretreated Oil palm with ionic liquid particularly 1-ethyl-3-methylimidazolium diethyl phosphate	Laccase from Trametes sp. Y120 having mediator HBT	Degradation of lignin content up to 35%	Material obtained having maximum content of cellulose	Financie et al. (2016)
Lignocellulosic feedstock of wheat straw	Laccases from *T. versicolor* with mediator HBT	Enhanced enzymatic hydrolysis	35% upturn glucose yield	Heap et al. (2014)
Silage corn stover	Laccases from *T. versicolor* and HBT as mediator	Side chain of lignin get oxidized	7% enhanced cellulose hydrolysis	Heap (2014)
Pretreated Cotton gin waste with a repeated combination of liquid hot water plus ultra-sonication	Laccase from *Cerrena unicolor* along with mediator 3,5-dimethoxy-4-hydroxybenzonitrile	Round about 15% lignin is lost	Glucose yield enhanced by 23% while 31% rose in ethanol yield	Placido et al. (2013)
Crushed eucalypt wood	Laccase from *T. villosa* in combination with mediator HBT	About 48% of lignin content was removed	61% escalation in glucose production	Gutiérrez et al. (2012)
Pretreated Phoenix dactylifera mild stuff left-over	HBT mediator in combination with laccase from *T. versicolor*	Modified lignin by subsequent decrease in hydrolytic enzymes binding	Sugar production increased	Al-Zuhair et al. (2013)

Moilanen et al. (2014) demonstrated that pretreated spruce LCB when subjected to LMS treatment (laccases from *T. hirsute* with ABTS, TEMPO, and HBT mediators) brought out increased enzymatic hydrolysis, i.e., 49% in case of TEMPO and 54% with ABTS, while HBT mediator showed no effect. Another study stated 32% and 48% delignification of elephant grass and eucalypt with improved sugar yield of 12% and 61% respectively by treating them with laccases from *T. villosa* in combination with HBT mediator, followed by alkaline extraction (Gutiérrez et al., 2012). Furthermore, the use of LMS (*P. cinnabarinus* laccase with HBT mediator) also caused 37% delignification and 60% proliferation in sugar yield after enzymatic hydrolysis (Rencoret et al., 2016) by oxidizing lignin phenolic structures (Flórez Pardo et al., 2015).

Along with delignification, enzymatic hydrolysis can also be enhanced by LMS as one study reported 375.9 mg/g glucose yield as a result of enzymatic hydrolysis by performing LMS of pretreated wheat straw (Deng et al., 2019). Likewise, Moilanen et al. (2014) also reported a 19% increase in enzymatic hydrolysis due to LMS pretreatment of LCB. While, Heap and his colleagues evidenced that LMS of (laccases from *T. versicolor* with mediator HBT) pretreated LCB of wheat straw boosted 35% glucose yield, followed by the extraction of alkaline peroxide (Heap et al., 2014).

## 8 Laccases for detoxification and delignification in a lignocellulose-based bio refinery

Delignification and detoxification of lignocellulosic raw materials via laccases are definite, potent, cost-effective, energy-efficient, and eco-friendly processes besides increasing the fermentation and saccharification yields by diminishing the inhibitory compounds (Hashmi et al., 2016). Lesser inhibitory content in pretreated biomass leads to higher product yield in a short period by limiting the side reactions and implementing moderate conditions to proceed with the reaction (Moreno et al., 2015).

There is a huge need to explore new commercial laccases with the potential to detoxify and delignify raw materials at once via biorefinery. Moreno et al. (2016b) assessed MetZyme^®^ to escalate the saccharification and ethanol fermentation of pretreated LCB, bringing about a 5% proliferation in sugar yield. The keystone of biorefinery impression is the maximal usage of bioresources in a tenable way, accompanied by a negligible impact on the environment (Moncada B et al., 2016). Biorefineries are responsible for the production of abundant and inexpensive fuel, along with the production of valuable compounds (Chowdhury et al., 2018). For this purpose, microbial laccases are significant as they support the outcomes and synthesis of new compounds from LCB. They also catalyze the reticulation of phenolic compounds in lignin-holding biomass and also avoid the usage of lethal synthetic adhesives (Shanmugam et al., 2020; Malhotra and Suman, 2021). Lacasses-mediated de-polymerization of lignin into numerous aromatic and phenolic composites is an auspicious methodology, and it has upgraded the status of laccases all across the globe.

## 9 Genetic engineering of fermenting microbes for laccase production

Remarkable advancements have been made to make delignification and detoxification processes more efficient. Genetic engineering is one of the most encouraging schemes as it genetically modifies fermenting microbes to produce laccases in compensation for traditional ways of manually adding laccases in pretreated biomass. For this purpose, several genes are also being overexpressed resulting in improved tolerance of microbes to inhibitory composites. Among microbes, yeast is the most proficient as it is easily manipulated, requires cost-effective culturing methods, and displays speedy growth along with swift and smooth genetic manipulation (Shukla, 2019; Aza et al., 2021). Therefore, many genetically engineered yeast strains are being developed these days (Antošová and Sychrová, 2016). Random mutagenesis along with heterologous or homologous genes is usually overexpressed to increase yeast forbearance to inhibitory complexes. Dehydrogenase plus reductase genes are widely overexpressed to enhance the detoxification capability of yeast for HMF and furfural inhibitors. In the past few years, different strains have been developed to make this process more efficient. A gene named ADH6p (NADPH-dependent alcohol dehydrogenase enzyme) was developed to improve the HMF as well as furfural reduction in yeast particularly in *S. cerevisiae*. Research highlighted that the overexpression of the ADH6p gene resulted in increased ethanol production during the fermentation process of spruce hydrolysate. Another study stated that upregulation of the ZWF1 gene from pentose phosphate shunt in yeast caused its shoot-up tolerance for furfuran byproducts. This tolerance is elucidated due to the pervasiveness of the pentose phosphate shunt alongside other pathways which increase the NADPH level intracellularly (Moreno et al., 2015).

The increased bioethanol production from raw materials is the prime purpose of genetic engineering these days. Larsson et al. (2001) genetically modified the strain of *S. cerevisiae* with laccases extracted from *T. versicolor*. The PKG1 promotor was used as a control to proliferate its resistance to phenolic inhibitors present in the LCB. The results showed that overexpression of homologous t-SNARE Sso2p can be used to raise the laccase activity twice. Genetic engineering favors detoxification, improved saccharification yield and ethanolic fermentation courses. Then contributes significantly to minimizing the procedure’s cost by eliminating the steps of detoxification and laccases production. Zhang et al. (2012) described the improvement in saccharification yield on account of modified *Trichoderma reesei* from the laccases of *Trametes*. Genetically engineered species also raise laccase activity, as Mate and his colleagues reported 34,000 folds increase in laccase activity by genetically engineering *S. cerevisiae* with laccases from basidiomycete (Mate et al., 2010; Antošová and Sychrová, 2016). These outcomes have corroborated the effectiveness of genetic engineering for proficient bioethanol production by altering the huge array of microorganisms.

## 10 Conclusion

Worldwide, nations have executed, characterized, or are running after the improvement of bio-economy techniques. The requirements for practical and manageable bio-based procedures support a huge section of the bioeconomy perspective. Laccase practices in biofuel production presently lean on the conception of lignocellulosic biomass degradation by laccases and the laccase mediator system. Both approaches are exceptionally efficient in the oxidative alteration of lignin besides increasing the hydrolysis yield from pretreated biomass. Nonetheless, enzyme production cost and expensive mediators (synthetic) are the contemporary challenges for practicing laccases in valorizing LCB, which can be resolved through microbial culture screening and genetic engineering. Several efforts are being instigated for immobilizing and engineering microbes for active laccases production, yet; the technique obliges more attention to achieve a superior future for biorefinery industries. There is also a huge need to uncover natural, eco-friendly, latest, and cheap mediators, which thrust the applications of these enzymes on the industrial scale, for enhanced biofuel production. Furthermore, a detailed study must be done on intracellular, yellow, white, thermostable, and alkaline laccases besides concentrating on the physiological and thermodynamic properties of laccases. Moreover, efforts should be carried out to devise cost-effective methods for the active production of the enzyme, which will aid in commercializing laccases all across the globe. Future research should focus on optimizing laccase production through genetic engineering for increasing yields and robustness. Additionally, exploring laccase synergy with other enzymes and developing efficient co-expression systems in microorganisms could lead to improved delignification and overall bioethanol conversion efficiency. This line of research has the potential to revolutionize bioethanol production by making it a more commercial and sustainable alternative fuel source.
